# Metabolomics Analysis Reveals the Differential Metabolites and Establishes the Therapeutic Effect Prediction Nomogram Among CP/CPPS Patients Who Respond or Do Not Respond to LiST

**DOI:** 10.3389/fimmu.2022.953403

**Published:** 2022-07-14

**Authors:** Jialin Meng, Chen Jin, Jiawei Li, Song Zhang, Meng Zhang, Zongyao Hao, Xianguo Chen, Zhengyao Song, Li Zhang, Chaozhao Liang

**Affiliations:** Department of Urology, The First Affiliated Hospital of Anhui Medical University; Institute of Urology & Anhui Province Key Laboratory of Genitourinary Diseases, Anhui Medical University, Hefei, China

**Keywords:** low-intensity extracorporeal shock wave therapy, chronic prostatitis/chronic pelvic pain syndrome, metabolomics, nomogram, therapy

## Abstract

**Objective:**

Low-intensity shockwave therapy (LiST) has been applied in the clinical treatment of chronic prostatitis/chronic pelvic pain syndrome (CP/CPPS), but few studies have focused on the prediction of its therapeutic effect before treatment.

**Methods:**

Seventy-five CP/CPPS patients from our institute between July 2020 and May 2021 were enrolled and received 3 Hz, 0.25 mJ/mm^2^ LiST once a week over the course of four weeks. The scores of the NIH-CPSI, IPSS questionnaire and demographic features before treatment were recorded. The plasma before LiST treatment was also collected, while liquid chromatography-tandem mass spectrometry was used to detect the metabolites. Least absolute shrinkage and selection operator (LASSO) regression analysis was employed to identify the prediction metabolites and generate the metabolism score. Receiver operating characteristic curves and calibration curves were drawn to assess the prediction accuracy of the nomogram.

**Results:**

Twelve metabolites were identified at incomparable levels before and after LiST treatment. The metabolism score generated by LASSO analysis presented a perfect prediction value (AUC: 0.848, 95% CI: 0.719-0.940) in the training cohort and further increased to 0.892 (95% CI: 0.802-0.983) on the nomogram, which accompanied with the NIH-CPSI scores and age. Similar results of the metabolism score (AUC: 0.732, 95% CI: 0.516-0.889) and total nomogram (AUC: 0.968, 95% CI: 0.909-1.000) were obtained in the testing cohort. Further enrichment of the 12 metabolites indicated that the glycine and serine metabolism pathway was involved in the LiST treatment.

**Conclusion:**

We used our system to accurately and quantitatively measure plasma metabolites and establish a predictive model to identify suitable patients for LiST treatment.

## Introduction

Chronic prostatitis/chronic pelvic pain syndrome (CP/CPPS) is the chronic disease of the prostate, and the CP/CPPS-like symptoms including repetitive episodes of pelvic pain, low urinary tract symptoms, mental disorders, and even sexual dysfunction (1). The prevalence of CP/CPPS is differs between countries and populations, which ranges from 8.4% to 25.3% worldwide (2, 3). The aetiology and pathogenesis of CP/CPPS is still perplexing, and in recent years, infection, defective function of urothelial, abnormal gut microbiota, psychosocial status, alcohol abuse, and lifestyle habits have been reported as risk factors (4–7). Immune system disorders, including the imbalanced infiltration of macrophages, Treg cells, and cytokines (8–11), have also been determined as the underlying causes of CP/CPPS.

There are many treatment approaches available in current clinical practice, including α-blockers, antibiotics and nonsteroidal anti-inflammatory medicines, M-receptor blockers, antidepressants, acupuncture, and physiotherapy (12). However, these treatments are only beneficial to part of CP/CPPS patients, so it is necessary to develop novel treatment methods, especially for patients with refractory CP/CPPS-like symptoms. Low-intensity shockwave therapy (LiST) is a novel non-invasive therapy that is gradually applied in the clinical treatment of several diseases (13, 14), including tendonitis, non-union fractures, plantar fasciitis and osteonecrosis, in addition to chronic wound healing in patients with diabetic and venous ulcers (15–17), because of its capacity to promote angiogenesis, regenerate nerves and reduce inflammation at a low-intensity level (18, 19).

LiST is a promising treatment for CP/CPPS patients. Saker et al. (20) reported that, according to a prospective randomized double-blind placebo-controlled clinical trial, 82.8% of patients had a ≥ 6-point decrease in the NIH-CPSI total score at the first follow-up visit after LiST. Another back-to-back clinical trial conducted by Kim et al. (21) reported similar results; patients who received LiST treatment met the clinical amelioration criteria when compared to the placebo group, including total, pain and quality of life scores recorded by the NIH-CPSI questionnaire. Recently, Mykoniatis et al. (22) summarized the recent studies and highlighted the efficacy and safety of LiST to CP/CPPS patients clinical treatment, provided the high level of evidence about the effectiveness of LiST. Our team also assessed the treatment effectiveness of LiST in 91 patients with CP/CPPS-like symptoms in a prior study, the effective rate after 4 rounds of treatment was approximately 51.65%, which increased to 68.18% after 8 rounds of treatment (23).

Considering the results in the literature regarding LiST treatment for patients with CP/CPPS-like symptoms, we should also remember that only some patients can benefit from LiST, therefore, it is necessary to identify the biomarkers and construct the prediction model to help the selection of patients who are suitable for the LiST treatment. Metabolomics is a qualitative and quantitative analysis of all metabolites in the peripheral blood, which can reflect the stable changes of internal environment that impacted by diseases (24, 25). CP/CPPS is a chronic inflammation associated disease, which always lasted more than three months, therefore, the detection of metabolites in peripheral blood can help to characteristic the internal environment status of patients with CP/CPPS-like symptoms. In the current study, we collected the patients with CP/CPPS-like symptoms and also received regular LiST treatment, conducted the comparison between responders and non-responders, to identify the prognostic metabolites which can help to select the suitable patients to receive LiST treatment, which is purposed to identify a new treatment strategy for patients with CP/CPPS-like symptoms.

## Patients and Methods

### Inclusion and Exclusion Criteria of Participants

From July 2020 to May 2021, seventy-five patients with CP/CPPS-like symptoms were enrolled to receive LiST at our institution. The enrolled criteria included the following: (1) > 18 years old and < 60 years old; (2) comply with the NIH classification (26), patient suffered from the pain or discomfort in the perineal or pelvic region for at least a three-month period within the last six months; (3) refractory CP/CPPS-like symptoms and failure of the standard treatment; (4) completion of 4 rounds of LiST treatments. The exclusion criteria were as follows: (1) current urinary tract infection; (2) diagnosed with benign prostatic hyperplasia; (3) prostate-specific antigen (PSA) > 4 ng/ml; (4) history of pelvic surgery; (5) history of heart disease; and (6) current use of anticoagulants. All patients were randomly assigned to the training cohort (50 patients) and testing cohort (25 patients).

### Collection of the Clinical Parameters

We recoded the following demographic features: age, body mass index (BMI), NIH-CPSI scores (27) for pain, urination and quality of life, IPSS scores for obstruction and irritation. According to the evaluation criteria of efficacy, a 6-point decrease in NIH-CPSI total score was regarded as the optimal threshold for predicting treatment response (28), and patients in different cohorts were further divided into the response group or the non-response group.

### LiST Treatment Protocols

For the clinical LiST treatment, we chose a low-intensity shockwave with a frequency of 3 Hz and an energy density of 0.25 mJ/mm^2^ based on prior studies (23, 29). Patients received the treatment once a week over the course of four weeks. After the digital rectal examination, approximately 2000 pulses were delivered obliquely to multiple areas of the perineum. The hand-held probe was placed on the perineum at an angle of 0°- 30° to the coronal plane and at an angle from 10° to 20° to the sagittal plane to treat the left and right lobes of the prostate gland, respectively. Each lobe received approximately 1000 pulses.

### Curative Effect and Safety Evaluation

We recorded the NIH-CPSI score of all enrolled patients evaluated before and after the LiST treatment (last time follow-up, one week after the 4^th^ treatment), a decline of six point was regarded as the response to LiST treatment (30). Each patient received the urinalysis at the last time follow-up. During the treatment period and the follow-up days, side effects such as subcutaneous bruising, ecchymosis, transient hematuria, and hemospermia were recorded by professional medical staff if occurred.

### Metabolite Profiling

We collected the blood plasma of each patient before LiST treatment. Whole blood was obtained, placed into a tube with ethylenediaminetetraacetic acid, and then centrifuged at 3000 rpm for 5 minutes. The supernatant was frozen at -80°C for metabonomic analysis. The metabolites in plasma were measured by liquid chromatography-tandem mass spectrometry (LC–MS/MS), and 11896 metabolites were obtained, among 707 named metabolites.

An ACQUITY UPLC I-Class system (Waters Corporation, Milford, USA) and VION IMS QTOF Mass spectrometer (Waters Corporation, Milford, USA) were used to illustrate the metabolic spectra in both ESI positive and ESI negative ion modes. The analysis of acquired LC–MS raw data was performed by progenesis QI software (Waters Corporation, Milford, USA) using the following parameters. The resulting matrix was further reduced by removing any peaks with a missing value (ion intensity=0) in more than 50% of samples. The internal standard was used for data quality control.

### Construction and Verification of the Nomogram

A total of 707 identified metabolites were enrolled for the least absolute shrinkage and selection operator (LASSO) regression, which was executed by the “*glmnet*” package. The minimum lambda value was defined as a cut-off point to minimize the mean cross-validated error. Subsequently, the metabolism score was calculated by the summation of the metabolite data multiplied by the corresponding index obtained from LASSO analysis. A nomogram was established by combining the statistically significant demographic indicators, clinical symptom scales, and plasma metabolites. Calibration performance was assessed by the calibration curve, which described the consistency of the predicted and observed events of response to LiST treatment. The Hosmer–Lemeshow test was performed to further evaluate the consistency between the predicted efficacy and the actual response rate (*P* > 0.05 was considered a good consistency). Additionally, the receiver operating characteristic (ROC) curve and the area under the curve (AUC) were calculated to evaluate the predictive discriminative ability of the nomogram.

### Statistical Analysis

Continuous variables conforming to a normal distribution are presented as the mean plus or minus standard deviation [mean ± standard deviation (SD)]; otherwise, they are presented as the median and range/interquartile range [median, interquartile range (IQR)]. Baseline comparisons between two cohorts were performed by independent sample t tests or Mann–Whitney U tests, and paired t tests were used to compare the differences before and after treatment. Categorical variables are expressed as frequencies and percentages (n, %), and the chi-square (χ^2^) test or Fisher’s test was used for variation analysis. All above analyses were conducted by IBM SPSS Statistics for Windows, version 25.0 (IBM Corp., Armonk, N.Y., USA), and R software, version 4.0.2. A two-tailed *P value* < 0.05 was considered significantly different.

## Results

### Baseline Characteristics of the Two Cohorts

First, we summarized the clinical characteristics of the patients in the training and testing cohorts (**Table 1**). All the 75 patients received four times LiST treatment and completed the follow-up. All the patients did not show the abnormal results in urinalysis before and after LiST treatment. In addition, no apparent adverse reactions such as hematuria, hemospermia, subcutaneous bruising, ecchymosis, etc., were observed during the four-week treatment and follow-up. To be brief, 50 patients (66.67%) were included in the training cohort, and 25 patients (33.33%) were randomly separated into the testing cohort. There was no significant difference in basic features in the two cohorts, including BMI (*P* = 0.096), educational level (*P* = 0.657), course of disease (*P* = 0.483), NIH-CPSI (*P* = 0.348), pain domain (*P* = 0.238), urination domain (*P* = 0.881), QoL domain (*P* = 0.263), IPSS (*P* = 0.479), irritation symptoms (*P* = 0.249), and obstruction symptoms (*P* = 0.879). Overall, the two cohorts were comparable. Although the age distribution appeared to be different in the two cohorts (*P* = 0.006), we adjusted it in the subsequent construction of the prognostic nomogram.

**Table 1:** Baseline characteristics of the training and testing cohort.

Parameters	Training (n = 50)	Testing (n = 25)	P-value	Test Statistics
Age, years (m, range)	35, 21-58	29, 22-53	0.006*	Z = -2.701
BMI, kg/m2 (mean ± SD)	24.42 ± 2.96	23.23 ± 2.55	0.090	t = 1.717
Education (n, %)			0.657	χ^2^ = 1.344
Junior and below	11, 22.00%	7, 28.00%		
High to undergraduate	36, 72.00%	18, 72.00%		
Graduate	3, 6.00%	0, 0.00%		
Course, months (m, range)	42 (3,480)	36 (3, 180)	0.483	t = -0.709
NIH-CPSI (mean ± SD)				
Total scores	25.56 ± 5.12	24.40 ± 4.80	0.348	t = 0.944
Pain domain	10.8 ± 2.94	10.00 ± 2.31	0.238	t = 1.19
Urination domain	5.02 ± 2.59	5.12 ± 2.95	0.881	t = -0.15
QoL domain	9.74 ± 1.61	9.28 ± 1.77	0.263	t = 1.127
IPSS (mean ± SD)				
Total scores	11.72 ± 6.98	12.96 ± 7.37	0.479	t = -0.712
Obstruction symptom	5.88 ± 4.36	7.24 ± 5.53	0.249	t = -1.162
Irritation symptom	5.84 ± 3.29	5.72 ± 3.06	0.879	t = 0.152

### The Different NIH-CPSI, IPSS, and Subgroup Scores Among the Response and Non-Response Groups in the Training Cohort

The efficacy and difference results of the response group (27 patients) and the non-response group (23 patients) in the training cohort are listed in detail in **Table 2**. In the response group, the NIH-CPSI scores (26.53 ± 4.82 vs. 15.41 ± 5.58, *P* < 0.001) and IPSS scores (11.89 ± 7.09 vs. 7.22 ± 4.85, *P* < 0.001) were significantly different before and after LiST treatment (*P* < 0.01), as well as the specific sub-domains, while the NIH-CPSI scores (24.43 ± 5.34 vs. 23.00 ± 6.72, *P* = 0.074) and IPSS scores (11.52 ± 7.01 vs. 9.83 ± 6.63, *P* = 0.058) showed no significant difference in the non-response group. We further compared the decrease in scores between the response and non-response subgroups. We revealed significant differences in NIH-CPSI score (11.11 ± 4.06 vs. 1.43 ± 3.67, *P* < 0.001) and IPSS (4.67 ± 5.33 vs. 1.70 ± 4.07, *P* = 0.034), as well as for the comparison of specific domains, such as the pain domain (6.00 ± 2.59 vs. 0.52 ± 3.19, *P* < 0.001), urination domain (2.48 ± 2.34 vs. 0.44 ± 1.44, *P* < 0.001) and QoL domain (2.63 ± 2.10 vs. 0.48 ± 1.56, *P* < 0.001) of the NIH-CPSI, and the obstruction symptoms (3.07 ± 3.68 vs. 1.04 ± 3.02, *P* = 0.040) of the IPSS.

**Table 2:** Scores of NIH-CPSI, IPSS, and their subgroup in training cohort.

Train corhot (n = 50)	Response (n = 27)			Non-response (n = 23)			P-value^R^	P-value^NR^	P-value^D^
	Baseline	Endpoint	Decline	Baseline	Endpoint	Decline			
NIH-CPSI	26.53 ± 4.82	15.41 ± 5.58	11.11 ± 4.06	24.43 ± 5.34	23.00 ± 6.72	1.43 ± 3.67	<0.001^*^	0.074	<0.001^*^
Pain domain	11.56 ± 2.31	5.56 ± 3.12	6.00 ± 2.59	9.91 ± 3.37	9.39 ± 4.15	0.52 ± 3.19	<0.001^*^	0.441	<0.001^*^
Urination domain	5.33 ± 2.84	2.85 ± 1.70	2.48 ± 2.34	4.65 ± 2.27	4.22 ± 2.39	0.44 ± 1.44	<0.001^*^	0.162	<0.001^*^
QoL domain	9.63 ± 1.45	7.00 ± 2.35	2.63 ± 2.10	9.87 ± 1.82	9.39 ± 2.06	0.48 ± 1.56	<0.001^*^	0.156	<0.001^*^
IPSS	11.89 ± 7.09	7.22 ± 4.85	4.67 ± 5.33	11.52 ± 7.01	9.83 ± 6.63	1.70 ± 4.07	<0.001^*^	0.058	0.034^*^
Obstruction symptom	5.89 ± 4.21	2.81 ± 3.01	3.07 ± 3.68	5.87 ± 4.64	4.83 ± 5.10	1.04 ± 3.02	<0.001^*^	0.112	0.040^*^
Irritation symptom	6.00 ± 3.52	4.41 ± 3.07	1.59 ± 2.26	5.65 ± 3.05	5.00 ± 2.88	0.65 ± 2.57	0.001^*^	0.236	0.175

^R^, Paired T-test of the Base line and Endpoint of Response group;

^NR^, Paired T-test of the Base line and Endpoint of Non-response group;

^D^, T-test of the decline points of Response and Non-response groups;

^*^, P < 0.05

### Generation of the Metabolism Score in the Training Cohort

**Figure 1:**
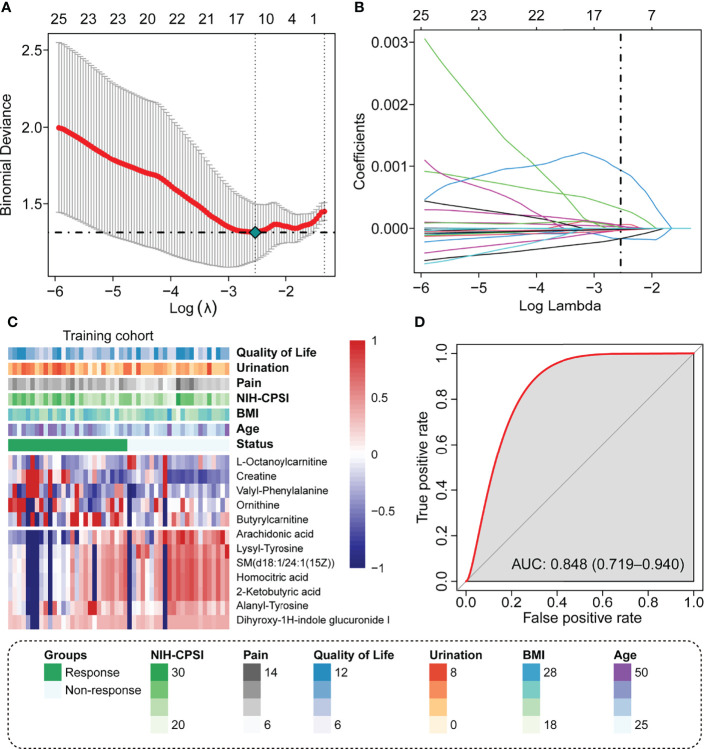
Identify the predicted metabolite response to the LiST treatment response. **(A)** The optimal tuning parameter (lambda) in the LASSO analysis selected with 10-fold cross-validation and one standard error rule; **(B)** LASSO coefficient profiles of the 25 metabolites; **(C)** Heatmap showing the distribution of 12 selected prediction metabolites and clinical features in the training cohort; **(D)** Prediction value of metabolism score assessed by the ROC curve.

A total of 707 recognizable metabolites were used for the LASSO analysis. With the minimal lambda value, 12 kinds of plasma metabolites were screened out (**Figures 1A, B**), including L-octanoylcarnitine, creatine, valyl-phenylalanine, ornithine, butyrylcarnitine, arachidonic acid, lysyl-tyrosine, SM(d18:1/24:1(15Z)), homocitric acid, 2-ketobutyric acid, alanyl-tyrosine, and dihyroxy-1H-indole glucuronide I. The values of the 12 metabolites in the responder and non-responders are displayed in **Figure 1C**, as well as the clinical scores from questionnaires. The metabolism score was calculated by the summation of the metabolite data multiplied by the corresponding index; details of the index are listed in **Supplementary Table 1**.

We observed that these patients who responded to LiST therapy had high plasma levels of L-octanoylcarnitine, creatine, valyl-phenylalanine, ornithine, and butyrylcarnitine, and these patients who did not respond to LiST therapy had high levels of arachidonic acid, lysyl-tyrosine, SM(d18:1/24:1(15Z)), homocitric acid, 2-ketobutyric acid, alanyl-tyrosine, and dihyroxy-1H-indole glucuronide I (**Figure 1C**). The basic information and molecular structure lists in **Supplementary Table 2**. The combined metabolism score was calculated as described in the methods section, and we revealed that the metabolism score presented a wonderful prognostic value to judge the clinical outcome of LiST (AUC: 0.848, 95% CI: 0.719-0.940, **Figure 1D**).

### Construction of the Nomogram in the Training Cohort

**Figure 2:**
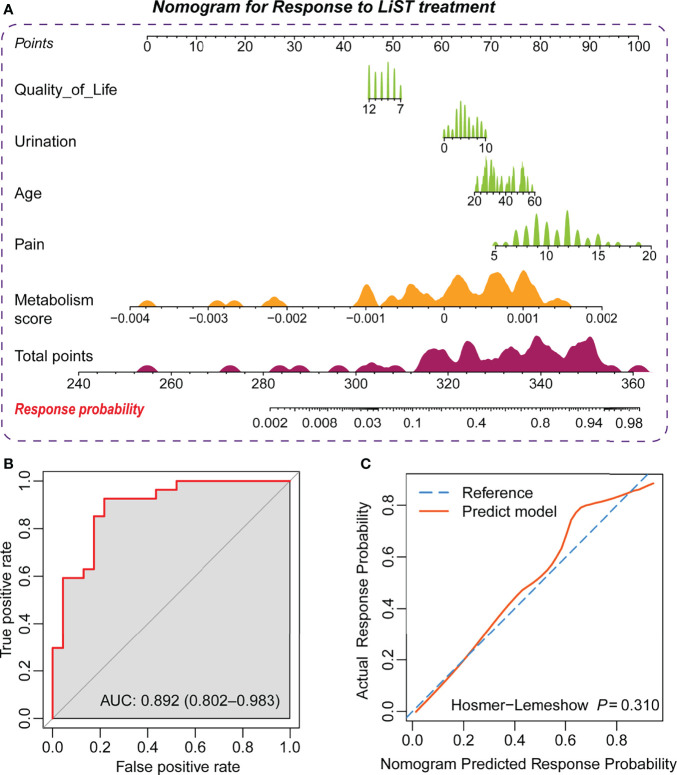
Establishment of the prediction nomogram for the LiST treatment response. **(A)** Nomogram showing weight of metabolism score, age, score of quality of life, urination, and pain from NIH-CPSI in training cohort; **(B)** ROC curve showing the prediction value of nomogram; **(C)** Calibration curve showing the comparison between actual response probability and nomogram predicted response probability.

To graphically represent the effect of each predictor on the efficacy in LiST and provide a more tangible interpretation of each predictor’s impact on the outcome, we established a nomogram in the training cohort, where age, pain domain, urination domain, QoL domain, and metabolic index score were also retained. Considering that not all IPPS subgroup scores had statistically significant differences, this predictor was not included in the model. Each independent predictor score was plotted into a straight line, and the scores for each factor were added together and positioned in the bottom line to evaluate the probability of response to the treatment of LiST in patients with CP/CPPS-like symptoms (**Figure 2A**). We verified the predictability and consistency of this nomogram. The combined points summarized by the nomogram presented a favourable prognostic value of efficient treatment, evaluated by the AUC value of 0.892, with the 95% CI ranging from 0.802 to 0.983 (**Figure 2B**). Meanwhile, we also conducted a calibration curve, which showed the high consistency of the predicted events to observed events, assessed *via* the Hosmer–Lemeshow test (*P* = 0.31, **Figure 2C**)

### Validation of the Nomogram in the Testing Cohort

**Figure 3:**
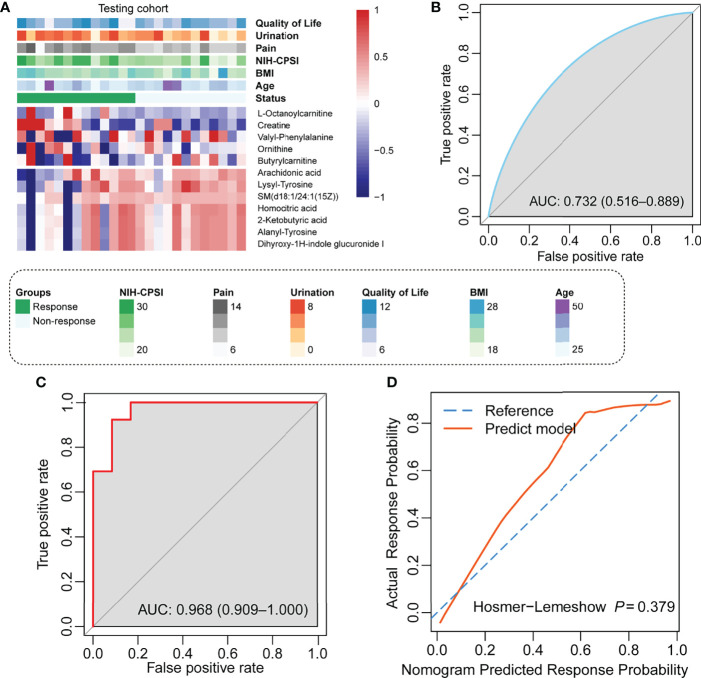
Validation of the prediction nomogram in the testing cohort. **(A)** Heatmap showing the distribution of 12 selected prediction metabolites and clinical features in the testing cohort; **(B)** ROC curve showing the prediction value of the metabolism score; **(C)** ROC curve showing the prediction value of the nomogram; **(D)** Calibration curve showing the comparison between actual response probability and nomogram-predicted response probability.

Similarly, we compared the NIH-CPSI, IPSS, and the sub-domains among responders and non-responders of LiST treatment in the testing cohort. The results were consistent with those findings in the training group, with significant differences in the decrease in scores between the response and non-response groups among the NIH-CPSI score and the sub-domains of the pain, urination and QoL domains (**Table 3**). In the testing cohort, the distribution of 12 plasma metabolites showed a consistent pattern compared to the training cohort (**Figure 3A**). We also calculated the metabolism score along with the formula obtained from the training cohort. The predictive value of the metabolism score in the testing cohort was evaluated by the AUC value, with a preferable AUC value of 0.732, and the 95% CI ranged from 0.516 to 0.889 (**Figure 3B**). Notably, the combined value from the nomogram plus clinical features showed a more accurate value in the efficacy prediction of LiST treatment, with an AUC and 95% CI of 0.968 (0.909-1.000) (**Figure 3C**). At the same time, the results of the calibration curve showed that the predicted results were relatively consistent with the actual results, with a Hosmer–Lemeshow statistical test *P* value of 0.379 (**Figure 3D**).

**Table 3:** Scores of NIH-CPSI, IPSS, and their subgroup in testing cohort.

Test cohort (n = 25)	Response (n = 13)			Non-response (n = 12)			P-value^R^	P-value^NR^	P-value^D^
	Baseline	Endpoint	Decline	Baseline	Endpoint	Decline			
NIH-CPSI	26.54 ± 4.43	15.15 ± 4.72	11.39 ± 4.48	22.08 ± 4.19	20.50 ± 3.29	1.58 ± 2.50	<0.001^*^	0.051	<0.001^*^
Pain domain	11.15 ± 2.38	5.69 ± 3.40	5.46 ± 3.67	8.75 ± 1.49	8.33 ± 2.35	0.42 ± 2.71	<0.001^*^	0.605	0.001^*^
Urination domain	6.08 ± 2.60	3.00 ± 163	3.08 ± 2.06	4.08 ± 3.06	3.50 ± 2.75	0.58 ± 1.51	<0.001^*^	0.206	0.002^*^
QoL domain	9.31 ± 2.21	6.46 ± 2.54	2.85 ± 2.12	9.25 ± 1.22	8.67 ± 1.07	0.58 ± 1.31	<0.001^*^	0.152	0.004^*^
IPSS	13.62 ± 8.25	6.00 ± 4.06	7.62 ± 5.75	12.25 ± 6.57	9.25 ± 5.74	3.00 ± 4.11	<0.001^*^	0.028^*^	0.032^*^
Obstruction symptom	7.92 ± 6.01	2.46 ± 1.45	5.46 ± 5.68	6.50 ± 5.13	4.33 ± 3.99	2.17 ± 3.64	0.005^*^	0.064	0.101
Irritation symptom	5.69 ± 3.55	3.54 ± 3.43	2.15 ± 2.08	5.75 ± 2.60	4.92 ± 2.68	0.83 ± 1.90	0.003^*^	0.157	0.111

^R^, Paired T-test of the Base line and Endpoint of Response group;

^NR^, Paired T-test of the Base line and Endpoint of Non-response group;

^D^, T-test of the decline points of Response and Non-response groups;

^*^, P < 0.05

All results show that the nomogram model established by the included predictor has good accuracy and stability and could be applied to evaluate the efficacy of LiST.

## Discussion

In the current study, we enrolled 75 patients who received sequential LiST treatment 4 times within a month. All these patients are with refractory CP/CPPS-like symptoms, which means that the standard treatment is ineffective for them. Patients were randomly separated into training and testing cohorts to establish and validate the metabolism score-based prediction nomogram. A total of 707 recognizable metabolites were enrolled in the LASSO regression analysis, and 12 metabolites were identified. The metabolism score was calculated along with the metabolic level and corresponding index. The scores obtained from the NIH-CPSI questionnaire and age were used to construct a prediction nomogram with a preferable prediction value and it was successfully validated. These results indicate that the metabolism score-based prediction nomogram is a promising tool used to select the patients CP/CPPS-like symptoms who will most likely benefit from LiST treatment.

CP/CPPS is regarded as a chronic disease marked by an imbalance of immunocytes and cytokines in peripheral blood. Meanwhile, researchers also revealed that only one third patients have the histological inflammation on prostate biopsy (31), the prostate local inflammation does not consistent with the severity of symptoms, and the IIIB category even does not show the evidence of inflammation (32, 33). Our previous study revealed increased proportions of central memory T cells, Th1, Th17 and Th22 cells, in the peripheral blood of CP/CPPS patients when compared with healthy controls (11). Currently, LiST is a widely used treatment for CP/CPPS patients, especially for refractory patients with recurrent clinical symptoms. The curative effectiveness of LiST for CP/CPPS might be based on its regulatory action on inflammatory-associated pathways or components. Feng et al. (34) demonstrated that LiST could degrade the expression of IL-1β, IL-6, TNF-α, and COX-2 in an EAP mouse model and reduce oxidative stress, inflammation, and pain *via* the PI3K/AKT/FOXO1 pathway. Jeon et al. (35) also reported that LiST can reduce the inflammation caused by CP/CPPS by degrading COX-2 in the microenvironment *via* the TLR4-NFκB-inhibiting pathway. Our team also revealed that LiST treatment can reduce the infiltration of total and degranulated mast cells around the prostate gland in a prior study (36). Identifying the potential mechanism of the method by which LiST modifies the clinical symptoms of CP/CPPS is shedding light; however, the predictors that determine whether patients can benefit from LiST are still unknown. Therefore, the current metabolism score-based prediction nomogram can help urologists determine which patients are suitable for LiST treatment.

Life activities in the cell are jointly undertaken by many genes, proteins, and small molecular metabolites, and the functional changes of upstream macromolecules will eventually be reflected at the metabolic level. Therefore, the metabolic group is downstream of the gene regulatory network and protein interaction network, providing terminal biological information. Metabolomics is a qualitative and quantitative analysis of all metabolites in an organism in a specific period, which has a stable effect (24, 25). CP/CPPS is a chronic inflammation associated disease, which always lasted more than three months, as compared to the urine or seminal fluid, the metabolites level in the blood plasma can reflect the constancy status of internal environment of patient in a long time of the past period. UPOINTS system (37) focuses on the different clinical symptoms and creates multimodal treatment plans based on their phenotype, the LiST might be more effective significant Urinary symptoms, infection-related symptoms and tenderness of skeletal muscles, therefore, the metabolites can help to identify the patients which are more suitable to receive the LiST treatment. Several studies report the predict function to disease of metabolites in plasm. Huang et al. reported an eight-metabolite index to predict the progress of mild cognitive impairment to Alzheimer’s disease, with the AUC value of 0.96. Cui et al. identified a six-metabolite model, including 12-HETE, Ceramide (d18:1/18:2 OH), L-palmitoylcarnitine, LysoPC(18:0), PI(36:2), and SM(d18:1/16:2), to predict the angina recurrence to patients who received percutaneous coronary intervention, with the predict accuracy of 89%. The current study is the first study which focus on the treatment effective prediction of CP/CPPS patients received LiST treatment, plasma metabolites are stable factors to reflect the internal environment for CP/CPPS patients, we constructed the metabolites-based LiST response prediction nomogram, and also validated in an independent cohort. These new findings can not only help the clinical practice of the selection of appropriate patients to receive the LiST treatment, but also support the potential mechanism study of how CP/CPPS altering and impacting patients and serve for the development of new drugs. In addition, several limitations need to be illuminated in the future study. First, multi-centre study with more CP/CPPS patients are needed to validate the prediction accuracy of the metabolism score and nomogram; Second, along with the sample size calculation formula provided by Riley et al.’s study (38), the minimal size to validate the prognostic nomogram is 383 patients, we will ongoing to collect patients receive LiST treatment, to further validate the prognostic nomogram. Third, the underlying mechanisms of how these metabolites reflect or indicate the LiST treatment outcome should be clarified.

## Conclusion

In summary, we used our system to accurately and quantitatively measure plasma metabolites to establish a predictive model to identify suitable patients for LiST treatment. Further validation cohorts are still needed to confirm our new findings and to overcome the limitations. Nevertheless, plasma metabolites as part of an integrative predictive model for CP/CPPS patients may act as a useful non-invasive biomarker that can help select CP/CPPS patients who can benefit from LiST treatment.

## Data Availability Statement

The datasets presented in this study can be found in MetaboLights (https://www.ebi.ac.uk/metabolights/), with the accession number: MTBLS5179.

## Ethics Statement

The studies involving human participants were reviewed and approved by Ethical approval was obtained from the Ethics Committee of the First Affiliated Hospital of Anhui Medical University (PJ-2021-15-38). Written informed consent for participation was not required for this study in accordance with the national legislation and the institutional requirements.

## Author Contributions

Conception and design: JM, MZ, and CL. Acquisition of data: JL, SZ and CJ. Analysis and interpretation of data: JM, JL and ZS. Statistical analysis: JM, JL, and XC. Drafting of the manuscript: JM, MZ, CJ, ZS, and LZ. Administrative, technical, or material support: XC, CL, and LZ. Supervision: XC, ZH, CL, and LZ. Final Approval of Manuscript: All the authors. All authors contributed to the article and approved the submitted version.

## Funding

This work was supported by the National Natural Science Foundation of China (82170787, 81870519, 81970597), the Scientific Research Foundation of the Institute for Translational Medicine of Anhui Province (No. 2017ZHYX02), the Key Project of Provincial Natural Science Research Project of Anhui Colleges (No. KJ2019A0278), The Supporting Project for Distinguished Young Scholar of Anhui Colleges (No. gxyqZD2019018), Joint Project of Urology and Hygiene Toxicology (2021lcxk013). The funder was not involved in the study design, collection, analysis, interpretation of data, the writing of this article or the decision to submit it for publication.

## Conflict of Interest

Wikkon Precision Technologies Ltd, Shenzhen, China provided the device and funded the project.

The remaining authors declare that the research was conducted in the absence of any commercial or financial relationships that could be construed as a potential conflict of interest.​

## Publisher’s Note

All claims expressed in this article are solely those of the authors and do not necessarily represent those of their affiliated organizations, or those of the publisher, the editors and the reviewers. Any product that may be evaluated in this article, or claim that may be made by its manufacturer, is not guaranteed or endorsed by the publisher.

## Supplementary Material

Supplementary Table 1 The metabolites and corresponding index obtain from LASSO analysis.

Supplementary Table 2 Detail information of the metabolites.
