# Supplementary material for: Metabolomics Analysis Reveals the Differential Metabolites and Establishes the Therapeutic Effect Prediction Nomogram Among CP/CPPS Patients Who Respond or Do Not Respond to LiST
Source: Front Immunol. 2022 Jul 14;13:953403. doi: 10.3389/fimmu.2022.953403 (PMC9332892; doi:10.3389/fimmu.2022.953403)
Supplement: Supplementary Table 1 — The metabolites and corresponding index obtain from LASSO analysis. [file Table_1.docx]

**Supplementary Table 1. The metabolites and corresponding index obtain from LASSO analysis.**

| metabolite | index |
| --- | --- |
| (Intercept) | -1.76E-02 |
| 2-Ketobutyric acid | -7.93E-04 |
| Alanyl-Tyrosine | -7.22E-04 |
| Arachidonic acid | -1.49E-05 |
| Butyrylcarnitine | 6.34E-06 |
| Creatine | 1.87E-06 |
| Dihyroxy-1H-indole glucuronide I | -1.68E-05 |
| Homocitric acid | 9.85E-05 |
| L-Octanoylcarnitine | -6.52E-07 |
| Lysyl-Tyrosine | 6.72E-06 |
| Ornithine | 2.25E-05 |
| SM(d18:1/24:1(15Z)) | -1.24E-07 |
| Valyl-Phenylalanine | 1.12E-06 |
