# Supplementary material for: Metabolomics Analysis Reveals the Differential Metabolites and Establishes the Therapeutic Effect Prediction Nomogram Among CP/CPPS Patients Who Respond or Do Not Respond to LiST
Source: Front Immunol. 2022 Jul 14;13:953403. doi: 10.3389/fimmu.2022.953403 (PMC9332892; doi:10.3389/fimmu.2022.953403)
Supplement: Supplementary Table 2 — Detail information of the metabolites. [file Table_2.docx]

**Supplementary Table 2. Detail information of the metabolites.**

| **Metabolite** | **HMDB** | **PubChem** | **KEGG** | **Structure** |
| --- | --- | --- | --- | --- |
| L-Octanoylcarnitine | HMDB0000791 | 11953814 | C02838 | 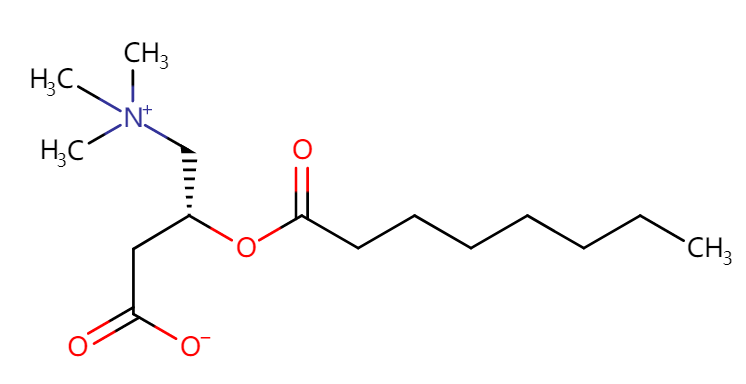 |
| Creatine | HMDB0000064 | 586 | C00300 | 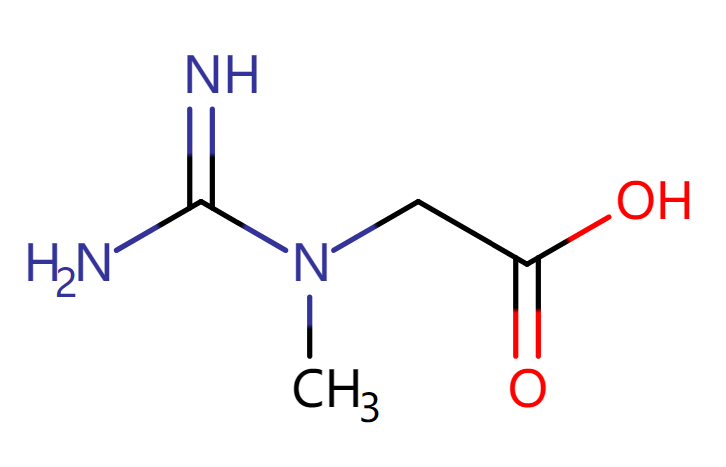 |
| Valyl-Phenylalanine | HMDB0029134 | 6993120 | NA | 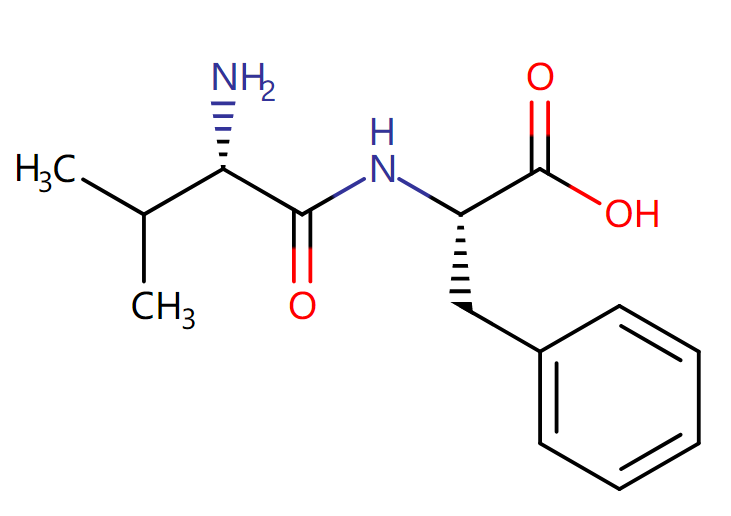 |
| Ornithine | HMDB0000214 | 6262 | C00077 | 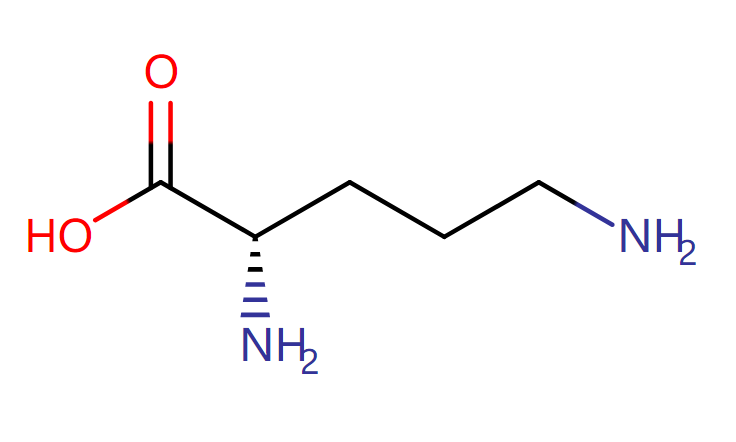 |
| Butyrylcarnitine | HMDB0002013 | 439829 | C02862 | 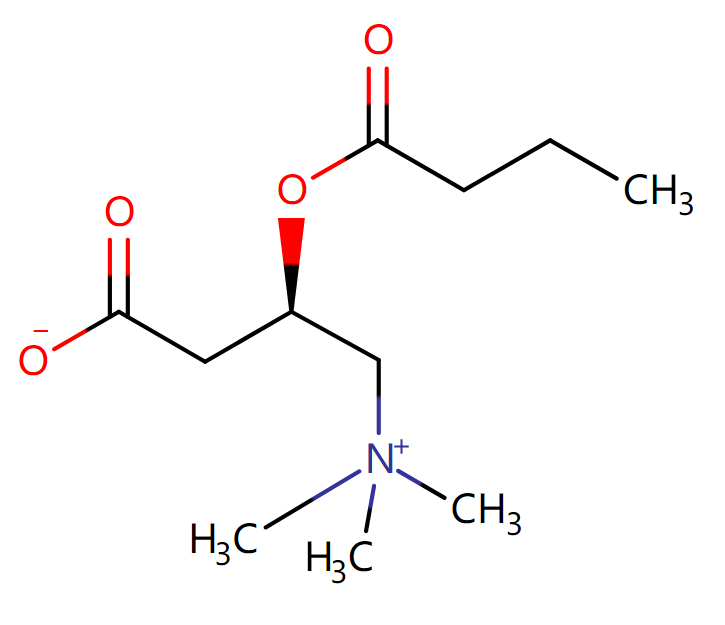 |
| Arachidonic acid | HMDB0001043 | 444899 | C00219 | 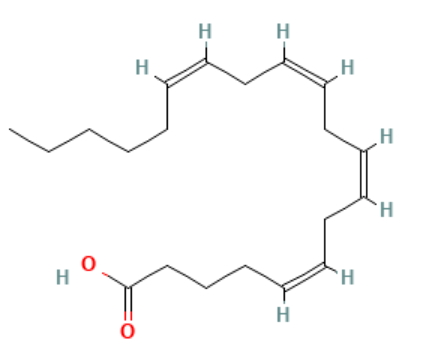 |
| Lysyl-Tyrosine | HMDB0028963 | 7021820 | NA | 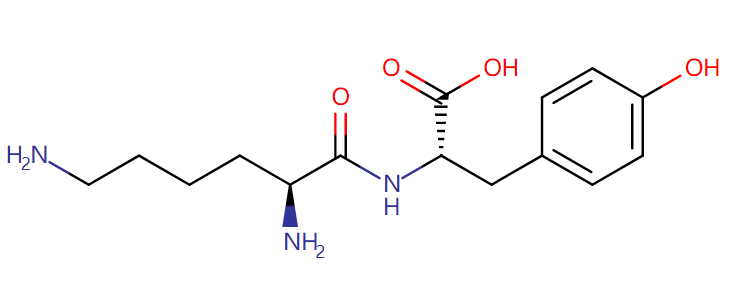 |
| SM(d18:1/24:1(15Z)) | HMDB0012107 | 44260126 | C00550 | 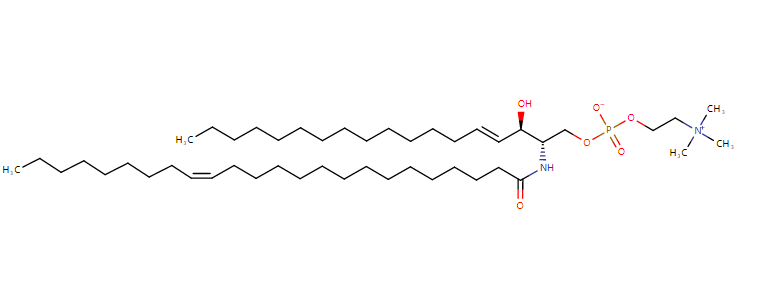 |
| Homocitric acid | HMDB0003518 | 439459 | C01251 | 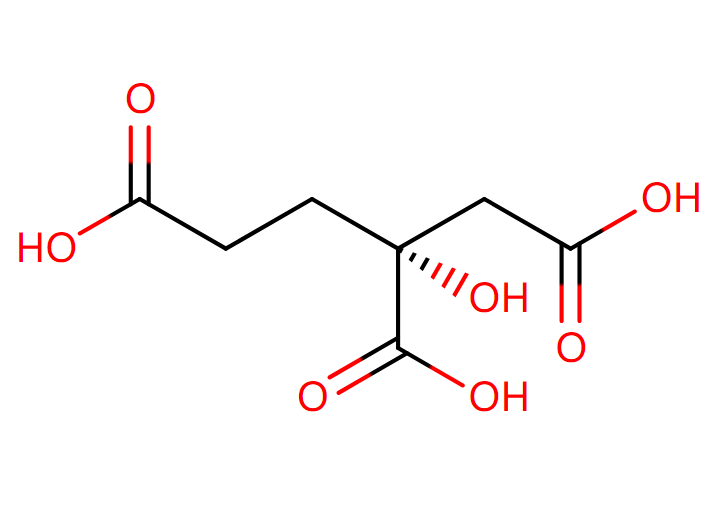 |
| 2-Ketobutyric acid | HMDB0000005 | 58 | C00109 | 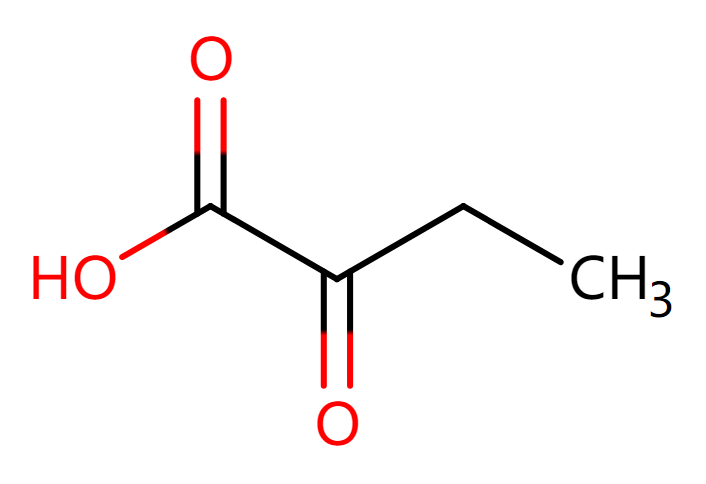 |
| Alanyl-Tyrosine | HMDB0028699 | 92946 | NA | 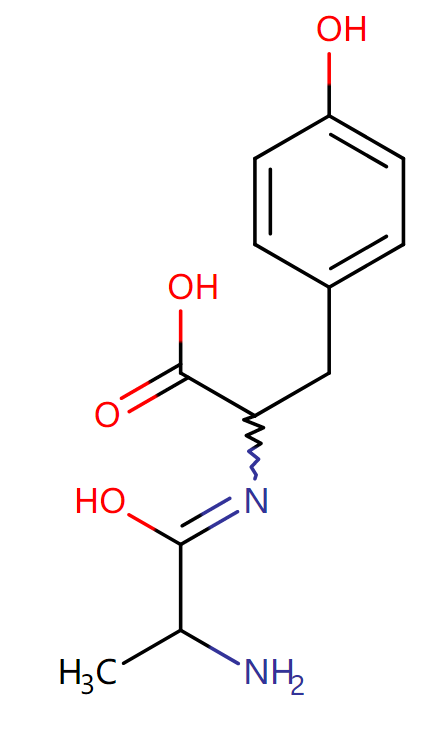 |
| Dihyroxy-1H-indole glucuronide I | HMDB0059997 | 124202061 | NA | 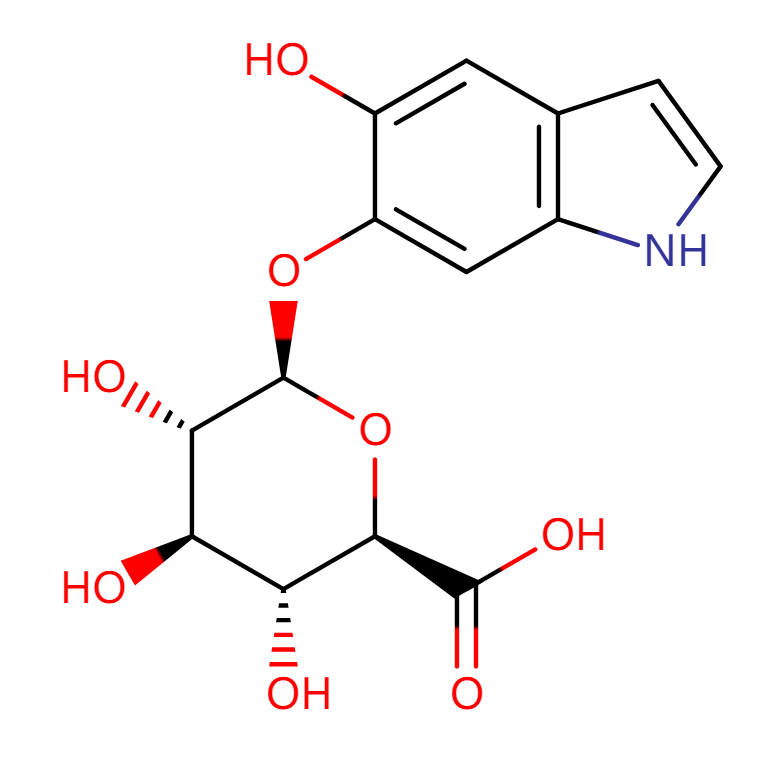 |
